# The relationship between maternal mental health and communication skills in children in Shiraz, Iran

**DOI:** 10.4178/epih.e2019035

**Published:** 2019-07-19

**Authors:** Najmeh Maharlouei, Hossein Alibeigi, Abbas Rezaianzadeh, Pedram Keshavarz, Hadi Raeisi Shahraki, Hamid Nemati, Kamran B. lankarani

**Affiliations:** 1 Health Policy Research Center, Institute of Health, Shiraz University of Medical Sciences, Shiraz, Iran; 2 Student Research Committee, Shiraz University of Medical Sciences, Shiraz, Iran; 3 Colorectal Research Center, Shiraz University of Medical Sciences, Shiraz, Iran; 4 Department of Radiology, Medical Imaging Research Center, Shiraz University of Medical Sciences, Shiraz, Iran; 5 Department of Epidemiology and Biostatistics, Faculty of Health, Shahrekord University of Medical Sciences, Shahrekord, Iran; 6 Shiraz Neuroscience Research Center, Shiraz University of Medical Sciences, Shiraz, Iran; 7 Health Policy Research Center, Institute of Health, Shiraz University of Medical Sciences, Shiraz, Iran

**Keywords:** Mental health, Child development, Questionnaire, Iran

## Abstract

**OBJECTIVES:**

Child development is a significant issue in global public health, and maternal mental health (MMH) can have a remarkable effect on children’s development of communication skills. We aimed to investigate the association between MMH and communication skills in a sample of Iranian children.

**METHODS:**

This study was conducted in Shiraz, Iran during 2016. In total, 640 mothers who lived in Shiraz and were registered in the Fars Birth Cohort (FBC) study were invited to attend the FBC clinic with their children. A trained physician evaluated MMH using the General Health Questionnaire (GHQ). Additionally, a trained nurse assessed the children’s communication development status using the Ages and Stages Questionnaire for 60-month old children.

**RESULTS:**

The majority of the mothers were homemakers (82.8%) and had high school diplomas (38.9%). The mothers’ mean age was 33.7±4.6 years. Seventy-nine (12.3%) children had delayed communication skills, but no significant association was found between children’s communication skills and the mothers’ total GHQ score (p=0.43). In total, 493 mothers (77.0%) had abnormal somatic symptoms, 497 (77.7%) had abnormal anxiety/insomnia, 337 (52.7%) had social dysfunction, and 232 (36.3%) suffered from depression. Logistic regression indicated that after adjusting for confounders, the odds of delayed communication skills were 3-fold higher among the children of mothers with abnormal somatic symptoms than among other children (p=0.01).

**CONCLUSIONS:**

The study results confirmed that MMH had a significant impact on children’s communication skills. Moreover, maternal abnormal somatic symptoms exerted the strongest impact on the development of communication skills in 5-yearold children.

## INTRODUCTION

Children’s health and development are significant issues in global public health [1]. Evidence has indicated that early child development is a critical period of life impacted by genetics, in utero development, and maternal demographic features during the prenatal and postnatal periods [2]. However, the importance of maternal mental health (MMH) during pregnancy and afterwards, when the baby is rapidly developing and growing, has been underestimated in many studies [3-5]. MMH strongly affects children’s growth and poor MMH can have adverse impacts on a child’s communication skills [6]. Maternal psychological problems have been recognized as a significant risk factor for delays in child development [7]. MMH abnormalities may result from incomplete resource allocation, a lack of specialized expertise, weak healthcare systems, and poor knowledge of MMH. Hence, eliminating such obstacles might be effective in improving MMH [8]. Furthermore, MMH problems may cause defects in all aspects of children’s communication skills, resulting in inefficient time allocation on the part of physicians and especially nurses. Children with communication problems need child health nurses who, in turn, must ask mothers for help and resources. According to a health cost-effectiveness analysis, these efforts may not be worth the money and time [9]. Today, improvements in MMH and a decrease in the number of children with communication deficits have led to lower related healthcare costs. Therefore, child health nurses can provide services in other areas of healthcare [10].

In a recently published study on the determinants of exclusive breastfeeding, the authors mentioned that cohort studies should be conducted to identify robust causal relationships [11]. Numerous psychological reports using the developmental origins of health and disease model have studied the causes of mental problems and their associations with individuals’ attitudes in the fundamental phases of life [12,13]. However, few published studies from Iran have evaluated the determinants of children’s communication skills based on the Ages and Stages Questionnaire (ASQ). Thus, the present study aimed to investigate MMH and its relationship with children’s communication skills in a sample of Iranian children and to assess the effect of proper MMH on the need for child health nurses. The results of this study will help increase the understanding of the role of MMH in childhood communication skills.

## MATERIALS AND METHODS

This study was conducted in Shiraz, Iran during 2016 to assess the association between mothers’ mental status and their children’s communication skills. In total, 640 mothers who had given birth to a child during 2011, lived in Shiraz, and were registered in the Fars Birth Cohort (FBC) study [13] were called through their phone numbers recorded in the FBC data bank. We decided to call the mothers registered in the FBC study because they were trained to record all details regarding the health issues of their children and themselves. Because both MMH and children’s communication skills could be affected by stressors since birth, we had to consider numerous confounders. Hence, after defining the study objectives, the mothers were invited to attend the FBC clinic with their children who had been registered in the FBC study. As mentioned above, to reduce recall bias, we asked the mother to bring their notebook to the interview session. We also used the FBC study data bank on mothers’ history of smoking during pregnancy, as well as their history of stillbirth, abortion, and having any child with a congenital anomaly and/or a chronic illness to cross-reference the information provided by mothers. However, we excluded registered children from other cities of in Fars Province, as Shiraz is the fifth most populous city in Iran, with stressors and facilities that are markedly different from those of smaller cities and towns, which could meaningfully impact both MMH and children’s communication skills.

The mothers were interviewed by a trained medical doctor who had worked in the FBC clinic since the beginning of the FBC study. The children were also interviewed by a trained nurse in order to evaluate their communication skills. Both MMH status and children’s communication skills were checked at the same session.

### Data gathering instrument

#### Maternal checklists

The checklists contained the parents’ demographic information, including educational level and occupation. In addition, the General Health Questionnaire (GHQ) was used to evaluate MMH. This questionnaire included 28 questions (GHQ-28), and has been validated in Iran by previous studies [14,15]. This questionnaire is a psychiatric screening instrument for mental disorders designed by Goldberg & Hillier [16]. The GHQ was translated into Persian, the official language of Iran, which is comprehensible to virtually all Iranians. This questionnaire has an estimated sensitivity of 84.7%, specificity of 93.8%, and overall misclassification rate of 8.2% [17]. The validity and reliability of this instrument for the Iranian population were confirmed by Noorbala & Mohammad [17]. The standard GHQ-28 consists of 4 subscales, each containing 7 questions as follows: (I) somatic symptoms (items 1-7), (II) anxiety/insomnia (items 8-14), (III) social dysfunction (items 15-21), and (IV) severe depression (items 22-28). All questions in the GHQ have a 4-point scoring system with responses including “almost always,” “usually,” and “rarely.” A simple Likert scale is used for scoring the instrument (3, 2, 1, and 0). A total score of 23 or more and/or a subscale score of 6 or more was considered to indicate the presence of mental disorder(s) in the study population [18]. Considering the importance of the mothers’ history of psychiatric disorders, they were asked for a detailed medication history, including the name, dosage, and duration of medication use since the last follow-up conducted 3 years ago to reduce recall bias. Based on the data recorded since the beginning of the FBC study (2011), the mothers were considered to have used psychiatric medications if they were currently taking medications or had taken medications for more than 6 months since they had given birth to their children. The mothers were also asked about other factors affecting MMH, including their history of infertility (primary or secondary), abortion, stillbirth, and child death; the presence of any children with specific illnesses/anomalies; and their perceived economic and social status. Moreover, the previously recorded data were used to determine the duration of marriage, maternity leave, and history of smoking and medication consumption by the mothers during pregnancy and puerperium.

#### Children’s information

The children’s communication development was assessed using the ASQ for 60-month old children [19]. The ASQ has been revealed to function well as an assessment in children with various risk factors. As a parent reporting tool, it presents a significant opportunity for parents to communicate about any concerns they have regarding their child’s development. The original ASQ has been proven to be reliable and valid with a total sensitivity of 75% and specificity of 86% in detecting developmental delays [19,20], and it has been validated in Iran [14,15].

The children were interviewed by a trained nurse in the presence of their mothers in a private room. The children’s past medical history was also considered since it could affect their development. The children were considered to have delayed communication skills if they were 2 standard deviations below the cut-off point provided by Yaghini et al. [14] and Soltani et al. [15].

### Statistical methods

All statistical analyses were performed using SPSS version 23.0 (IBM Corp., Armonk, NY, USA). The independent t-test was used to compare the delayed and non-delayed groups with respect to quantitative variables. Additionally, associations between qualitative variables and the study groups were assessed using the chi-square or Fisher exact test as appropriate. Variables with p-values <0.20 in the univariate analysis were entered into the logistic regression model and backward elimination (alpha-to-remove=0.10) was implemented. The p-values <0.05 were considered to indicate statistical significance.

### Ethics statement

This study was approved by the Ethics Committee of Shiraz University Medical Sciences (IR.SUMS.MED.RED.1397.371). The mothers were informed of the confidentiality of their information. They were also reassured that they did not have to answer the questions if they did not feel comfortable doing so.

## RESULTS

In total, 640 mothers attended the FBC clinic for this study. The majority of the mothers were homemakers (82.8%), had high school diplomas (38.9%), and classified themselves as belonging to the moderate category of social (89.1%) and economic (81.9%) status. Their mean age and duration of marriage were 33.7±4.6 years and 11.1±4.9 years, respectively. Their mean number of children was 1.7±0.7 per woman and their mean monthly household incomes was US$457.1±401.1.

Among the study participants, 200 (31.3%), 4 (0.6%), and 3 (0.5%) had a history of abortion, child death, and children with some abnormalities, respectively. In addition, 52 mothers (8.1%) reported the presence of severe psychiatric disease (Tables 1 and 2). As shown in Table 2, 420 mothers (65.6%) were categorized as abnormal group in terms of the total GHQ score. Furthermore, 493 mothers (77.0%) had abnormal somatic symptoms, 497 (77.7%) had abnormal anxiety/insomnia, 337 (52.7%) had social dysfunction, and 232 (36.3%) suffered from severe depression.

Of the 640 children under investigation, 79 (12.4%) had delayed communication skills. The parents’ educational levels were significantly associated with their children’s communication skills (p< 0.001). Indeed, the proportion of delayed communication skills was significantly higher among the children of mothers with abnormal somatic symptoms than others (14.4% vs. 5.4%, p=0.004). However, no significant association was found between communication skills and the mothers’ total GHQ score (p=0.43) (Table 3).

The results of the logistic regression model are summarized in Table 4. The results indicate that after adjusting for the confounders, the odds of delayed communication skills were 3-fold higher among the children of mothers with abnormal somatic symptoms than among other children (p=0.01).

## DISCUSSION

The study results indicated that MMH had a major effect on children’s communication skills, which could cause healthcare personnel, particularly nurses, to become less involved with children’s deficient developmental skills. Although previous studies have revealed a relationship between MMH and communication problems in children, this was the first study to evaluate the association between MMH and communication skills assessed using the ASQ in Iranian children.

Generally, MMH problems are associated with children’s development [3]. Hence, it is essential to be aware the relationship between MMH and children’s development during and after pregnancy. MMH is essential for accomplishing at least 5 of the World Health Organization’s 8 Millennium Development Goals, namely promoting gender equality and empowering women, reducing child mortality, achieving universal primary education, improving maternal health, and reducing poverty [21]. Moreover, understanding how MMH problems early in childhood may affect children’s communication skills may yield critical information for preventing the onset of anomalies in children [22]. A prior study demonstrated the need for understanding the prevalence and determinants of children’s mental health in South Asia, where about half of the population has been estimated to be under 18 years old [23].

Overall, about two third of mothers participated in this study suffered from MMH problems, which was significantly higher than the previously documented rate (approximately 35%) of MMH problems in the Iranian general population [24,25]. Caring for MMH is a general way of addressing mothers’ physical, spiritual, and psychological health. Simultaneously, healthcare providers, including physicians and nurses, are responsible for treating and relieving patients’ and parents’ pain and pressure. Therefore, one of the purposes of this study was to determine how improvements in MMH could reduce the number of children with communication problems. This may lead to lower healthcare costs and a reduced demand for coverage by nurses and physicians in other healthcare fields. Other reports have shown the need for comprehensive care in healthcare to help mothers’ well-being [26].

The current study findings demonstrated a strong relationship between MMH and communication skills in 5-year-old children. Specifically, 26.7% of the children with communication delays had mothers with mental health abnormalities. Similar results were also obtained in other studies investigating this issue [17,18]. Furthermore, the mothers’ socio-demographic features, including age and educational level, were correlated with the children’s developmental status, with lower maternal educational level and higher age associated with an increased risk of mental problems in children. These results are in agreement with those reported by Koutra et al. [12]. Children living in families with MMH problems need care that focuses on protecting them against growth problems, including greater social support, more social relationships, and fewer responsibilities, all of which can have a considerable impact on the development of their communication skills [27].

The present study had some limitations, the first of which was the completion of the ASQ by a trained nurse, which might have caused children not to feel comfortable answering the questions. Another study limitation was that fathers’ mental health status was not considered when adjusting for factors associated with children’s communication skills. Furthermore, this study was conducted among participants from Shiraz, and the findings therefore cannot be extrapolated to all Iranian children.

The results of this study confirmed that MMH was a significant factor affecting children’s communication skills. Moreover, maternal abnormal somatic symptoms exerted the strongest impact on development of communication skills in 5-year-old children. Therefore, the timely detection of MMH abnormalities could prevent delays in communication skills in children.

## Figures and Tables

**Table 1.** Demographic characteristics of the mothers who participated in all three interview phases

Characteristics	n (%)	Characteristics	n (%)
Education level		Husband’s education level	
Below diploma	162 (25.3)	Below diploma	193 (30.2)
Diploma	249 (38.9)	Diploma	233 (36.4)
Academic	229 (35.8)	Academic	214 (33.4)
Mother’s job		Husband’s job	
Homemaker	530 (82.8)	Jobless	22 (3.4)
Part-time	32 (5.0)	Part-time	25 (3.9)
Full-time	53 (8.3)	Full-time	214 (33.4)
Two-shift	25 (3.9)	Two-shift	379 (59.2)
Perceived economic status		Perceived social status	
Low	13 (2.0)	Low	46 (7.2)
Middle	524 (81.9)	Middle	570 (89.1)
High	103 (16.1)	High	24 (3.8)
History of child death		History of stillbirth	
No	636 (99.4)	No	637 (99.5)
Yes	4 (0.6)	Yes	3 (0.5)
History of abortion		Presence of children with specific illnesses^1^	
No	440 (68.8)	No	499 (78.0)
Yes	200 (31.3)	Yes	141 (22.0)
Mother’s history of smoking		Presence of children with congenital anomalies	
No	363 (56.7)	No	637 (99.5)
Yes	277 (43.3)	Yes	3 (0.5)

1 Down syndrome, polymyositis, and autism.

**Table 2.** Frequency distribution of mothers according to GHQ items

Characteristics	n (%)
History of psychiatric medication^1^	
No	588 (91.9)
Yes	52 (8.1)
GHQ total^2^	
Mean±SD	30.6±15.1
Normal	220 (34.4)
Abnormal	420 (65.6)
GHQ I^3^	
Mean±SD	9.46±4.85
Normal	147 (23.0)
Abnormal	493 (77.0)
GHQ II^4^	
Mean±SD	10.1±5.20
Normal	143 (22.3)
Abnormal	497 (77.7)
GHQ III^5^	
Mean±SD	6.22±3.42
Normal	303 (47.3)
Abnormal	337 (52.7)
GHQ IV^6^	
Mean±SD	4.82±4.97
Normal	408 (63.8)
Abnormal	232 (36.3)

GHQ, General Health Questionnaire; SD, standard deviation.

1 The medications were prescribed by a physician.

2 GHQ total: GHQ-28 (items 1-28).

3 GHQ I: somatic symptoms (items 1-7).

4 GHQ II: anxiety/insomnia (items 8-14).

5 GHQ III: social dysfunction (items 15-21).

6 GHQ IV: severe depression (items 22-28).

**Table 3.** The frequency distribution of the mothers’ demographic characteristics and children’s communication skills

Characteristics	Communication skills		p-value
	Not delayed (n=561)	Delayed (n=79)	
Education level			<0.001
Below diploma	139 (85.8)	23 (14.2)	
Diploma	244 (98.0)	5 (2.0)	
Academic	178 (77.7)	51 (22.3)	
Husband’s education level			<0.001
Below diploma	169 (87.6)	68 (12.4)	
Diploma	232 (99.6)	1 (0.4)	
Academic	160 (74.8)	54 (25.2)	
Mother’s occupation			0.02
Homemaker	473 (89.2)	57 (10.8)	
Part-time	23 (71.9)	9 (28.1)	
Full-time	44 (83.0)	9 (17.0)	
Two-shift	21 (84.0)	4 (16.0)	
Father’s occupation			0.07
Jobless	20 (90.9)	2 (9.1)	
Part-time	19 (76.0)	6 (24.0)	
Full-time	181 (84.6)	33 (15.4)	
Two-shift	341 (73.9)	38 (10.0)	
Perceived social status			0.86
Low	11 (84.6)	2 (15.4)	
Middle	461 (88.0)	63 (12.0)	
High	89 (86.4)	14 (13.6)	
Perceived economic status			0.07
Low	36 (78.3)	10 (21.7)	
Middle	502 (88.1)	68 (11.9)	
High	23 (95.8)	1 (4.2)	
Child death			0.99
No	557 (87.6)	79 (12.4)	
Yes	4 (100)	0 (0.0)	
Stillbirth			0.99
No	558 (87.6)	79 (12.4)	
Yes	3 (100)	0 (0.0)	
Abortion			0.10
No	392 (89.1)	48 (10.9)	
Yes	169 (84.5)	31 (15.5)	
Mother’s history of smoking			0.60
No	316 (87.1)	47 (12.9)	
Yes	245 (88.4)	32 (11.6)	
Children with specific illnesses^1^			0.32
No	434 (87.0)	65 (13.0)	
Yes	127 (90.1)	14 (9.9)	
Psychiatric drugs consumption			0.11
No	519 (88.3)	69 (11.7)	
Yes	42 (80.8)	10 (19.2)	
Presence of children with congenital anomalies			0.99
No	558 (87.6)	79 (12.4)	
Yes	3 (100)	0 (0.0)	
Marriage duration, mean±SD (yr)	11.1±4.9	11.3±4.5	0.76
No. of children, mean±SD (n)	1.7±0.7	1.8±0.8	0.25
Monthly household income (US$)	456.9±407.6	458.7±354.0	0.97
General Health Questionnaire (GHQ) item			
GHQ total^2^			0.43
Normal	196 (89.1)	24 (10.9)	
Abnormal	356 (86.9)	55 (13.1)	
GHQ I^3^			0.004
Normal	139 (94.6)	8 (5.4)	
Abnormal	422 (85.6)	71 (14.4)	
GHQ II^4^			0.63
Normal	127 (88.8)	16 (11.2)	
Abnormal	434 (87.3)	63 (12.7)	
GHQ III^5^			0.53
Normal	263 (86.8)	40 (13.2)	
Abnormal	298 (88.4)	39 (11.6)	
GHQ IV^6^			0.51
Normal	355 (87.0)	53 (13.0)	
Abnormal	206 (88.8)	26 (11.2)	

Values are presented as number (%). SD, standard deviation.

1 Down syndrome, polymyositis, and autism.

2 GHQ total: GHQ-28 (items 1-28).

3 GHQ I: somatic symptoms (items 1-7).

4 GHQ II: anxiety/insomnia (items 8-14).

5 GHQ III: social dysfunction (items 15-21).

6 GHQ IV: severe depression (items 22-28).

**Table 4.** The results of logistic regression model for delayed communication skills

Characteristics		OR (95% CI)	p-value
Education level	Below diploma	1.00 (reference)	
	Diploma	0.13 (0.04, 0.41)	<0.001
	Academic	1.06 (0.42, 2.70)	0.90
Husband’s education level	Below diploma	1.00 (reference)	
	Diploma	0.04 (0.01, 0.36)	0.003
	Academic	2.73 (1.09, 6.81)	0.03
Economic status	Low	1.00 (reference)	
	Middle	5.18 (0.65, 41.21)	0.12
	High	10.88 (1.17, 101.09)	0.04
GHQ I^1^	Normal	1.00 (reference)	
	Abnormal	3.01 (1.34, 6.76)	0.01

OR, odds ratio; CI, confidence interval; GHQ, General Health Questionnaire.

1 GHQ I: somatic symptoms (items 1-7).
